# Promoting a Healthy Diet in Young Adults: The Role of Nutrition Labelling

**DOI:** 10.3390/nu10101335

**Published:** 2018-09-20

**Authors:** Zehra Buyuktuncer, Aylin Ayaz, Damla Dedebayraktar, Elif Inan-Eroglu, Basma Ellahi, Halit Tanju Besler

**Affiliations:** 1 Department of Nutrition and Dietetics, Faculty of Health Sciences, Hacettepe University, Ankara 06100, Turkey; baylin@hacettepe.edu.tr (A.A.); damla.yilmaz@hacettepe.edu.tr (D.D.); elif.inan@hacettepe.edu.tr (E.I.-E.); halit.besler@emu.edu.tr (H.T.B.); 2 Faculty of Health and Social Care, University of Chester, Chester CH1 4BJ, UK; b.ellahi@chester.ac.uk; 3 Faculty of Health Sciences, Department of Nutrition and Dietetics, Eastern Mediterranean University, Famagusta 99520, North Cyprus

**Keywords:** nutrition facts label, healthy eating index, young adults, dietary intake

## Abstract

The use of the nutrition facts label has been associated with healthy eating behaviors for adults. However, the relationship between nutrition facts label use and overall diet quality is not well known in young adults, a vulnerable group that acquire lifelong eating behaviors during this period of life. This study aimed to assess if the use of information on the nutrition facts label is associated with a higher diet quality in young adults. In this cross-sectional study, 958 university students aged 18–34 years were recruited. Nutrition facts label use was recorded. Dietary intake was assessed using 24-h dietary recall. Healthy Eating Index-2005 (HEI-2005) scores were calculated. HEI-2005 score was significantly associated with using nutrition facts label (*p* < 0.001). The mean total HEI-2005 score was 60.7 ± 10.11, 62.4 ± 11.43 and 67.1 ± 12.23 respectively for never, sometimes and everytime users of nutrition facts label (*p* < 0.001). Sub-group scores of HEI-2005 for total fruits, whole fruits, total vegetables, whole grains, milk, oils, saturated fat, and calories from solid fat, alcohol and added sugar (SoFAAS) were significantly higher in regular nutrition facts label users (*p* < 0.05, for each). This study showed that young adults who regularly use the nutrition facts label have a higher diet quality.

## 1. Introduction

The nutrition facts label, mostly found on processed foods, could be a cost-effective tool for communicating nutrition information to consumers at the point of purchase to help them make healthy food choices. It provides consumers with information about the energy and nutrient content of food and beverages, and thus, it provides a better understanding of foods purchased and consumed. Since both the consumption of processed foods and the prevalence of overweight and obesity have been increased in particular among young adults, the provision of more detailed nutrition information arose in this age group [1,2,3,4]. From a public health perspective, nutrition facts labels can help consumers to choose healthy foods and acquire healthy eating habits by providing nutrition information [5,6].

Studies on nutrition facts label use have been mainly conducted in high-income countries; nutrition facts label use in low or middle-income countries is not well known. However, citizens of low or middle-income countries can also take advantage of using the nutrition facts label as an efficient nutrition education tool to choose a better diet and healthy lifestyle [7].

Although food labelling is mandatory in most countries, the implementation of nutrition labelling varies from country to country. The use of nutrition facts labels has become mandatory in the US in 1990 with the Nutrition Labelling and Education Act (NLEA) [8]; and in the European Union in 2011 with the EU Regulation N. 1169/2011 [9]. In Turkey, food labelling is regulated by the Turkish Food Codex Food Labelling and Consumer Information Regulation, which was adapted from EU regulation by the Ministry of Food, Agriculture and Livestock [10]. According to this law, nutrition labelling is mandatory, and it must include information on energy, fat, saturated fat, carbohydrate, sugar, protein, salt, and trans-fat content of the products. Despite this regulation, our previous research showed that nutrition facts label use was reported by 72.4% of Turkish consumers [11].

Several studies have reported on the determinants of nutrition facts label use and how well the information is understood. These determinants are age, gender, level of education, health status, health and nutrition knowledge, household size, level of income (economic status), ethnicity, marital status and occupation, all of which have a relationship with nutrition facts label use and understanding [12,13,14,15,16,17,18,19,20,21,22]. Specific groups of consumers, such as young adults, can be targeted to develop use of the nutrition facts label for encouraging acquisition of healthy food preferences.

The transition period from high school to university is challenging for many young adults, and is one that is characterized by developing routines, habits, and preferences—many of which persist throughout adulthood [23]. This period is typically classified for young adults by a transition from eating with their parents at home to one where they plan and prepare their own meals at their new accommodation [24]. Adopting healthy dietary practices during this transitional period might affect consumption throughout adulthood, thus reducing the risk of chronic disease later in life [25]. However, university students have often been reported to adopt unfavorable dietary habits including lower consumption of fruits, vegetables, whole grains and legumes, meats and fish; higher consumption of fast food, sugar and alcohol during their studies [23,26,27,28,29,30,31]. Moreover, many young adults gain weight over the course of their university years, particularly during their first year [31,32]. Although previous studies found evidence that nutrition facts label use is associated with reduced fat, sugar and overall energy intake [33,34]; increased consumption of fruits and vegetables [35], and higher intake of fiber, vitamin C and iron [12,33], there is still much to learn about the relationship between nutrition facts label use and overall diet quality among young adults.

Our previous study showed that the frequency of nutrition facts label use was much lower in young adults compared to other adult groups [11]. It was believed that encouraging nutrition facts label use in young adults might increase their diet quality and help them to develop healthy eating behavior during this stage of the lifespan which, crucially, could be retained into later adulthood. However, the relationship between nutrition facts label use and overall diet quality measured by healthy eating index has not been studied in young adults. Therefore, this study hypothesized that young adults who read the nutrition facts label have a healthy diet. Against this background, this study aimed to assess the diet quality of young adults according to their usage of the nutrition facts label and its components.

## 2. Materials and Methods

### 2.1. Participants

This cross-sectional study was conducted on a sample of 647 (67.5%) female and 311 (32.5%) male university students, aged 18 to 34 years (mean 21.5 ± 1.86 years), attending undergraduate programs. The participants were randomly appointed by the administrative staff of each faculty. Written informed consent from volunteer participants was obtained prior to completing the study questionnaire. This study was conducted according to the guidelines laid down in the Declaration of Helsinki, and all procedures involving human subjects/patients were approved by the Hacettepe University Ethics Committee (HEK 12/412).

### 2.2. Questionnaire

A questionnaire, developed by the research team, assessing the use of information on food labels and, in particular, the nutrition facts label was administered by trained dietetics interns. The content validity of the questionnaire was measured by a pilot study with a sample size of 50 eligible participants in terms of inclusion and exclusion criteria. Some questions and response options were reworded based on the comments of participants in the pilot study. Statements about the use of information on the nutrition facts label (“How often do you pay attention to the calorie information on the food label when you buy food?”; and “How often do you pay attention to protein/fat/sugar information on the food label when you buy food?”) were answered on a 5-point Likert scale (ranging from “every time” to “never”). Since the use of five categories produced a large table with small sample size in some categories, in order to represent the findings more efficiently, categories were combined as follows: Participants who reported either of the “every time” or “almost every time” categories were categorized as “every time” and those who reported either of the “sometimes” or “rarely” categories were classified as “sometimes” during data analysis. Socio-demographic characteristics including gender, age, field, and year of study were also recorded. Moreover, anthropometric measurements including weight and height were taken by dietetics interns and body mass index (BMI) was calculated as weight (kg) divided by height squared (m^2^).

### 2.3. Dietary Quality

Dietary intake was assessed by trained dietetics interns using the 24-h dietary recall method. Diet quality was estimated with the Healthy Eating Index-2005 (HEI-2005) by using the analysis of 24-h dietary recall data. The components of the HEI-2005 represent all the major food groups; total fruit (scoring 5 points), total vegetables (5 points), total grains (5 points), milk including soy beverages (10 points) and meat and beans including poultry, fish, eggs, soybean products other than beverages, nuts, seeds, and legumes (10 points). Additional components represent whole fruit (i.e., forms other than juice) (5 points); dark green and orange vegetables and legumes (5 points); whole grains (5 points); oils (non-hydrogenated vegetable oils and oils in fish, nuts, and seeds) (10 points); saturated fat (10 points); sodium (10 points); and calories from solid fats, alcoholic beverages, and added sugars (20 points). The HEI-2005 score was categorized as “poor” (≤50), “needs improvement” (between 51 and 80) and “good” (>80) [36].

### 2.4. Statistical Analyses

Data were analyzed using the Statistical Package for the Social Sciences (SPSS), version 22.0. (SPSS Inc., Chicago, IL, USA) HEI-2005 scores were calculated using Microsoft Excel software (2007) (Microsoft Corporation, Redmond, WA, USA). Descriptive statistics were computed for general characteristics of participants and results were presented as frequency and percentage. The normal distribution of continuous variables was evaluated by the Shapiro-Wilk test. The results were presented as means with standard deviation and medians (minimum-maximum). The mean HEI-2005 scores across nutrition facts label use groups were compared by using Kruskal-Wallis test for non-parametric variables and one-way ANOVA for parametric variables. A multinomial logistic regression model was used to calculate odds ratios (ORs) and their 95% confidence intervals (CIs) to examine associations between diet quality and nutrition facts label use. For the multinomial logistic regression, the nutrition facts label use was categorized into two groups as “Yes” or “No”. Since the always users were considered as reference, only always users were categorized into “Yes”, and sometimes and never users were combined in the “No” group. This also provided an equable sample size in each group for statistical analysis, as the sample size of never users was small. Sex, age, living status and BMI variables were adjusted in multinomial logistic regression. The “good” HEI-2005 group was selected as the reference category. A *p* value < 0.05 was considered significant for all statistical tests.

## 3. Results

### 3.1. Characteristics of the Study Participants

Table 1 presents the general characteristics of the study sample. The mean age of the participants was 21.5 ± 1.85 years, mostly in a range of 18–24 years (95.9%). Many of the participants were attending 3rd and 4th year classes (33.6% and 35.6% respectively). While 40% of the participants were staying in dormitories or 30.7% of them were living with friends in a house, only 3.7% of them were living in a house alone. The mean BMI of participants was 21.98 ± 3.20 kg/m^2^. The majority (73.9%) of participants was within a normal body weight. More than half of the participants (54.7%) reported that they use the food label every time, whereas only 38.2% of the participants were recorded as everytime users of the nutrition facts label. The estimated HEI-2005 scores showed that the diet quality of most of the participants (77.3%) needed improvement.

### 3.2. HEI-2005 Scores and Nutrition Facts Label Use

The mean total HEI-2005 score was 60.7 ± 10.11, 62.4 ± 11.43 and 67.1 ± 12.23 for never, sometimes and everytime users of the nutrition facts label (*p* < 0.001), respectively. When the HEI-2005 sub-group scores of the participants were assessed based on the conditions of nutrition facts label use, the scores of total fruit, whole fruit, total vegetables, whole grains, milk, saturated fat, calories from solid fat, alcohol and added sugar sub-groups were found higher in “everytime” users of the nutrition facts label compared to “never” or “sometimes” users of the nutrition facts label (*p* < 0.05; for each). In the sub-group of oils, it was observed that the HEI-2005 scores of the participants, who never read the nutrition facts labels, were higher than the HEI-2005 scores of other groups (*p* < 0.05). However, the scores for dark green and orange vegetables and legumes, total grains, meat and beans and sodium sub-groups did not differ according to nutrition facts label use (*p* > 0.05; for each) (Table 2). 

### 3.3. HEI-2005 Scores and Components of the Nutrition Facts Label

In Table 3, the total HEI-2005 scores of the participants are presented, according to the use of different components of nutrition facts labels. It was found that participants, who always check the content of energy, protein, carbohydrate, sugar, fat, saturated fat, unsaturated fat, monounsaturated fat, polyunsaturated fat, omega-3, omega-6, trans-fat, cholesterol, fiber, salt/sodium, vitamins and minerals of product on nutrition facts labels, had higher total HEI-2005 scores than the participants who sometimes or never use the components of the nutrition facts label (*p* < 0.05; for each).

### 3.4. Association between HEI-2005 Groups and Nutrition Facts Label Use

A multinomial logistic regression analysis was performed to identify the association between nutrition facts label use and diet quality after adjusting related general characteristics (Table 4). The analyses showed that there was a significant association between the nutrition facts label use and total HEI-2005 score. The participants who need to improve HEI-2005 scores used the nutrition facts label 1.94 times less, and the participants who have poor HEI-2005 scores used the nutrition facts label 2.73 times less, when compared with the participants who have good HEI-2005 scores (*p* < 0.05, for each) (Table 4).

## 4. Discussion

The present study was undertaken to assess the relationship between nutrition facts label use and diet quality in young adults. It was found that nutrition facts label use was associated with a high HEI-2005 score. Our prior hypothesis was that participants who always use nutrition facts labels would be more likely to engage in the higher HEI-2005 scores, which was confirmed. Although this indicates that these constructs are positive determinants of dietary quality in this population, which is consistent with other studies among young adults in different countries, HEI-2005 was not used to assess overall diet quality in any of these studies [37,38].

It was found that statements for the use of the nutrition label differed in the conducted studies. While some studies found that more than half of the participants reported on using the nutrition facts label [4,38], other studies found that less participants reported on using the nutrition facts label [6,39]. In this study, it was also confirmed that 38.2% of the participants use the nutrition facts label every time.

In a previous study, high consumption of fruits and vegetables and low intake of fat were associated with nutrition facts label use [38]. Consistent with the study reported by Cooke and Papadaki (2014) [38], this study showed that the scores of the HEI-2005 sub-groups of total fruit, whole fruit and total vegetables were higher among participants who always read the nutrition facts labels. In addition to this, the scores for the sub-group of oils were lower in the participants who always read the nutrition facts labels compared to the other participants. However, contrary to this study, the mean intake of added sugar was found as lower among label users [38]. Healthier dietary habits among label users have also been demonstrated in other studies that include increased intakes of fiber, iron and vitamin C and reduced intakes of fat, sodium, cholesterol and total energy, as well as greater overall consumption of healthier foods such as fruits and vegetables [12,13,14,33,39,40,41,42,43,44]. Fitzgerald et al. (2008) [40] found that food label use in the case of high-fiber foods was associated with high consumption of fruits and vegetables. It was also stated that use of food labels to choose low sodium food was associated with decreased consumption of salty snacks. In another study, it was reported that food label users usually had healthier diets in terms of lower percentage of calories from fat and saturated fat, lower cholesterol and sodium intake, and higher fiber intake [41]. In addition to this, a laboratory-based study reported that availability of the nutrition facts label had a direct effect of decreasing total calorie intake of participants [45]. These findings suggest that the nutrition facts label could be an efficient tool to modify some dietary behaviors that can improve diet quality of individuals. Marietta et al. (1999) [46] reported that students who read nutrition labels were most interested in the fat content, calories and calories from fat. However, closer attention for a greater range of nutrients and food groups was obtained in this study. In addition to fat and energy content, it was found that the HEI-2005 scores of the participants, who always check the protein, carbohydrate, sugar, saturated fat, unsaturated fat, monounsaturated fat, polyunsaturated fat, omega-3, omega-6, trans-fat, cholesterol, fiber, salt/sodium, vitamin and mineral content of product on the nutrition facts labels, were higher than the scores of the others. Therefore, this study carried the results of the previous study one step forward, and concluded that not only fat content, calories or calories from fat but also the other components of the nutrition facts labels were checked by the university students with higher diet quality [46].

Another unique finding of this study was that nutrition facts label use was significantly associated with a higher HEI-2005 score after the adjustment of covariates including sex, age, living status and BMI. Graham et al. (2012) [37] also showed that frequent nutrition label users generally had healthy dietary behaviors such as higher consumption of fruits and vegetables compared to the infrequent nutrition label users. Moreover, it was reported that adults with good diet quality perceptions read nutrition facts labels more [47]. Miller et al. (2015) [48] also found that self-reported food label use is positively associated with dietary quality in an adult population. Although previous studies conducted on different populations had similar results with the results of our study, this study provides significant contributions to the existing literature as the findings of this study reflect the status of young adults, a vulnerable group in society in relation to acquiring healthy eating habits important for adult life.

Despite these strengths and contribution to the literature, several limitations to the present study are acknowledged. Firstly, it is important to note that this analysis is limited by the cross-sectional study design; thus, the study did not allow conclusions regarding causal relationship between nutrition facts label use and other parameters, only observations. Secondly, although the study has a large study population, there can be limitations for the generalization of the results because university students can be different from other young adult populations in terms of many ways including educational experiences, cooking skills, different environment and accessibility for healthy foods. Therefore, further studies with a big sample size from different environments representing the general population are required. It is also important to note that university settings used in different studies might be different from each other in terms of the student population and location. The university setting used in this research was located at the center of the country, with students from all over the country, and one of the largest university campuses with one of the biggest student number. However, a multi-center study including different university settings from different locations might reflect the profile of general university student population in the country more consistently. Finally, the assessment of diet was limited by the use of 24-h dietary recall. Since 24-h dietary recall represents short-term dietary intake, it might not be enough to assess the actual behavior, in particular food selection. A food frequency questionnaire might be a better tool to assess long-term food selection behavior; however, the lack of a validated food frequency questionnaire limits this for this study. On the other hand, the 24-h dietary recall method enabled a larger sample size to be obtained, as it was easy and quick to conduct. Despite the limitations, the findings of this study are worthy; as it is one of the first studies that report the relationship between nutrition facts label use and overall diet quality in university students, a sub-group of young adults.

## 5. Conclusions

Before developing new strategies to encourage the effective consumer use of the nutrition facts label, it was important to show the beneficial effects of nutrition facts label use on diet quality. Therefore, the findings of this study provide useful guidance for future nutrition interventions among university students, because it discusses that improving usage of nutrition facts labels might play a role in better diet quality. Also, well-planned nutrition education programs, which are designed to improve comprehension of nutrition principles and its reflection in food labels, including the explanation of the terms, statements, and symbols that appear on the labels, should incorporate the use of the nutrition facts labels in order to contribute to the future dietary habits and behavior of young adults. These aforementioned programs should start as early age as possible since the habits that acquired in childhood will more likely to remain in adulthood.

Information about the attitudes of young people, who often use packaged products and their use of nutrition facts labels will help our understanding and inform the development of public health nutrition education strategies. Using nutrition facts labels as a nutrition education tool could be an important health promotion policy objective in low and middle-income countries such as Turkey. Apart from nutrition education, steps to improve the standardized format of nutrition facts labels, both in content and visually such as a legible font size and clear presentation of expressions, terms, statements and symbols and to make nutrition facts labels simpler and more concise to promote better understanding of nutrition facts labels should be considered by policy makers. Future studies should be planned with broader and more representative samples while determining how the nutrition facts labels affect the diet quality using qualitative research to provide depth of understanding. Furthermore, experimental studies are also necessary to determine the relationship of nutrition facts labels on food choice, consequently diet quality.

## Figures and Tables

**Table 1:** General characteristics of the participants.

	N	% *
**Gender (*n* = 958)**		
Male	311	32.5
Female	647	67.5
**Age (*n* = 958)**		
18–24 years	919	95.9
25–34 years	39	4.1
**Class (*n* = 958)**		
1st year	71	7.4
2nd year	193	20.2
3rd year	322	33.6
4th year	341	35.6
5th year	31	3.2
**BMI (k/m^2^) (*n* = 958)**		
<18.5—underweight	99	10.3
18.5–24.9—normal	708	73.9
25.0–29.9—overweight	133	13.9
30.0–34.9—obese	18	1.9
**Food Label Use**		
Every time	524	54.7
Sometimes	299	31.2
Never	135	14.1
**Nutrition Facts Label Use**		
Every time	366	38.2
Sometimes	492	51.4
Never	100	10.4
**HEI-2005 classification**		
Good	95	9.9
Needs Improvement	740	77.3
Poor	123	12.8

* Percentages are given as column percentages.

**Table 2:** Total and sub-group HEI-2005 scores of participants according to their use of nutrition facts label.

Scores of HEI	Nutrition Facts Label Use						
	Never (*n* = 100)		Sometimes (*n* = 492)		Every Time (*n* = 366)		*p*
	X ± SD	Median	X ± SD	Median	X ± SD	Median	
		(Min–Max)		(Min–Max)		(Min–Max)	
Total fruit	2.4 ± 1.63	2.0 (0.0–5.0)	2.5 ± 1.60	2.0 (0.0–5.0)	2.7 ± 1.58	3.0 (0.0–5.0)	0.038 ^a^
Whole fruit	2.9 ± 1.58	3.0 (0.0–5.0)	3.0 ± 1.57	3.0 (0.0–5.0)	3.3 ± 1.58	3.0 (0.0–5.0)	0.011 ^a^
Total vegetables	2.0 ± 1.12	2.0 (1.0–5.0)	1.9 ± 1.03	2.0 (0.0–5.0)	2.1 ± 1.14	2.0 (0.0–5.0)	0.042 ^a^
Dark green and orange vegetables and legumes	3.3 ± 1.58	4.0 (1.0–5.0)	3.3 ± 1.53	3.0 (0.0–5.0)	3.5 ± 1.49	4.0 (0.0–5.0)	0.113 ^a^
Total grains	4.8 ± 0.74	5.0 (1.0–5.0)	4.6 ± 0.91	5.0 (1.0–5.0)	4.6 ± 0.95	5.0 (0.0–5.0)	0.051 ^a^
Whole grains	3.0 ± 1.81	3.0 (0.0–5.0)	2.9 ± 1.79	3.0 (1.0–5.0)	3.5 ± 1.76	5.0 (0.0–5.0)	<0.001 ^a^
Milk	3.6 ± 2.88	2.0 (0.0–10.0)	3.9 ± 2.67	3.0 (1.0–10.0)	4.6 ± 3.10	4.0 (1.0–10.0)	0.002 ^a^
Meat and beans	8.3 ± 2.96	10.0 (1.0–10.0)	8.3 ± 2.72	10.0 (1.0–10.0)	8.2 ± 2.74	10.0 (1.0–10.0)	0.654 ^a^
Oils	9.2 ± 2.08	10.0 (1.0–10.0)	8.7 ± 2.55	10.0 (0.0–10.0)	8.5 ± 2.52	10.0 (1.0–10.0)	0.006 ^a^
Saturated fat	3.2 ± 4.14	0.0 (0.0–10.0)	3.7 ± 4.30	0.0 (0.0–10.0)	4.6 ± 4.52	3.5 (0.0–10.0)	0.002 ^a^
Sodium	6.1 ± 3.35	8.0 (0.0–10.0)	6.2 ± 3.24	8.0 (0.0–10.0)	6.1 ± 3.25	8.0 (0.0–10.0)	0.946 ^a^
Calories from solid fat, alcohol and added sugar	12.1 ± 6.44	15.0 (0.0–20.0)	13.3 ± 7.17	15.0 (0.0–20.0)	15.5 ± 6.18	20.0 (0.0–20.0)	<0.001 ^a^
Total Healthy Eating Index Score	60.7 ± 10.11	61.0 (33.0–88.0)	62.4 ± 11.43	62.0 (28.0–94.0)	67.1 ± 12.23	67.0 (32.0–98.0)	<0.001 ^b^

^a^: *p* value was calculated by Kruskal-Wallis test; ^b^: *p* value was calculated by One-way ANOVA.

**Table 3:** Total HEI-2005 score of participants according to use of nutrition facts label components.

Components of Nutrition Facts Label	Total HEI Score According to Use of Nutrition Facts Label Components						
	Never		Sometimes		Every Time		*p* ^a^
	X ± SD	Median	X ± SD	Median	X ± SD	Median	
		(Min–Max)		(Min–Max)		(Min–Max)	
Nutrition Facts Label	60.7 ± 10.11	61.0 (33–88)	62.411.43 ±	62.0 (28–94)	67.2 ± 12.21	67.0 (32–98)	<0.001
Energy	61.3 ± 10.61	62.0 (32–88)	61.8 ± 11.33	62.0 (28–93)	66.3 ± 12.10	66.0 (32–98)	<0.001
Protein	62.1 ± 10.70	62.0 (33–91)	62.4 ± 11.84	62.0 (28–98)	67.0 ± 11.99	67.0 (32–96)	<0.001
Carbohydrate	62.4 ± 10.61	63.0 (32–88)	61.9 ± 11.70	61.0 (28–94)	67.2 ± 12.07	67.0 (32–98)	<0.001
Sugar	62.3 ± 10.35	63.0 (32–88)	61.9 ± 11.78	61.0 (28–94)	67.0 ± 12.13	67.0 (32–98)	<0.001
Fat	61.9 ± 10.42	63.0 (32–88)	62.0 ± 11.89	62.0 (28–94)	66.6 ± 12.01	67.0 (32–98)	<0.001
Saturated fat	62.0 ± 10.30	32.0 (33–91)	63.4 ± 12.23	63.0 (28–94)	67.2 ± 12.49	67.0 (32–98)	<0.001
Unsaturated fat	62.4 ± 10.49	63.0 (33–93)	63.3 ± 12.11	63.0 (28–94)	67.3 ± 12.64	67.0 (32–98)	<0.001
Monounsaturated fat	62.4 ± 10.64	62.0 (28–94)	63.9 ± 12.26	64.0 (32–94)	67.8 ± 12.99	68.0 (32–98)	<0.001
Polyunsaturated fat	62.4 ± 10.68	62.0 (28–94)	63.9 ± 12.26	64.0 (32–94)	67.9 ± 12.85	68.0 (32–98)	<0.001
Omega-3	62.3 ± 10.54	62.0 (28–94)	63.9 ± 12.15	64.0 (32–94)	67.7 ± 13.09	68.0 (32–98)	<0.001
Omega-6	62.4 ± 10.96	62.0 (28–94)	64.3 ± 12.24	64.0 (32–98)	67.1 ± 12.86	67.0 (32–96)	<0.001
Trans-fat	61.8 ± 10.54	61.0 (33–88)	63.4 ± 11.62	63.0 (32–94)	66.3 ± 12.67	66.0 (28–98)	<0.001
Cholesterol	61.2 ± 10.37	61.0 (33–91)	64.5 ± 12.41	64.0 (28–98)	66.6 ± 12.03	66.0 (39–96)	<0.001
Fiber	62.1 ± 10.66	62.0 (33–91)	63.4 ± 12.13	63.0 (28–94)	68.7 ± 12.36	69.0 (40–98)	<0.001
Salt/Sodium	61.7 ± 10.57	61.5 (32–92)	64.4 ± 12.16	64.5 (32–94)	67.1 ± 12.58	67.0 (28–98)	<0.001
Vitamins	62.3 ± 10.73	62.0 (28–93)	64.7 ± 12.00	64.0 (32–94)	64.9 ± 12.66	65.0 (32–98)	0.012
Minerals	62.1 ± 10.72	62.0 (28–88)	64.4 ± 12.21	64.0 (32–94)	65.7 ± 12.32	66.0 (32–98)	0.001

^a^: *p* values were calculated by one-way ANOVA test.

**Table 4:** Odds ratios of having high HEI-2005 score according to nutrition facts label use.

HEI-2005 Classification	Nutrition Facts Labels Use			
	Yes (*n* = 366)	No (*n* = 592)	OR	(95% CI)
Good	53 (14.5%)	42 (7.1%)	Ref.	
Needs Improvement	276 (75.4%)	464 (78.4%)	1.94 *	(1.24–3.04)
Poor	37 (10.1%)	86 (14.5%)	2.73 *	(1.52–4.90)

Multinomial logistic regression was used to calculate OR, Ref., reference category. CI indicates confidence interval; OR, odds ratio. Adjusted variables: Sex, age, BMI and living status * *p* < 0.05.
